# Breast cancer with scalp metastases: a case report

**DOI:** 10.1186/s13256-023-03928-8

**Published:** 2023-05-18

**Authors:** Ahmad M. Abdulraheem, Duha Naji, Ammar N. Al Heyasat, Mohammad Alhasan, Nidal M. Almasri, Raghad Odeh

**Affiliations:** 1 Department of Internal Medicine, King Hussein Cancer Center, Amman, Jordan; 2 School of Medicine/University of Jordan, Amman, Jordan; 3 School of Medicine/Jordan University of Science and Technology, Irbid, Jordan; 4 School of Medicine/Al-Balqa Applied University, Alsalt, Jordan; 5 Department of Pathology and Laboratory Medicine, King Hussein Cancer Center, Amman, Jordan

**Keywords:** Breast cancer, Scalp metastases, Immunohistochemistry

## Abstract

**Background:**

While breast cancer is the most common cancer in women, cutaneous metastases are rare in breast cancer. Additionally, scalp involvement in breast cancer metastasis is extremely rare. That being said, scalp lesions should always be thoroughly investigated to distinguish metastatic lesions from other neoplasms.

**Case presentation:**

A 47-year-old female Middle-Eastern patient presented with metastatic breast cancer in the lungs, bone, liver, and brain with no signs of multiple organ failure, in addition to cutaneous metastases, including the scalp. Between 2017 and 2022, she was managed through modified radical mastectomy, radiotherapy, and several lines of chemotherapy. She presented in September of 2022 with enlarging scalp nodules, which started developing 2 months prior to her presentation. Physical examination revealed firm, non-tender, and immobile skin lesions. Magnetic resonance imaging scan of the head showed soft tissue nodules in different sequences. A punch biopsy was taken from the largest scalp lesion and showed metastatic invasive ductal carcinoma. A panel of immunohistochemistry stains was applied, because a single specific marker for differentiating primary cutaneous adnexal tumors or other malignant neoplasms from breast cancer has not yet been identified. The panel showed positive estrogen receptor 95%, progesterone receptor 5%, negative human epidermal growth factor receptor 2, positive GATA binding protein 3, positive cytokeratin-7, negative P63, and negative KIT (CD117).

**Conclusion:**

Breast cancer metastases to the scalp are extremely uncommon. When a scalp metastasis is present, it might be the only symptomatic sign of disease progression or widespread metastatic lesions. However, such lesions warrant a comprehensive radiologic and pathologic workup to rule out other possibilities of skin pathologies, such as sebaceous skin adenocarcinoma as it effects the management plan.

## Introduction

Breast cancer is the most common cancer in females and the second leading cause of cancer death among females [1]. A study of global patterns of breast cancer incidence and mortality, conducted from the GLOBOCAN online database, showed an estimated 2.3 million new breast cancer cases among females in 2020, accounting for approximately 24.5% of all female cancer cases worldwide, and 685,000 deaths were recorded in 2020, which accounted for 15.5% of female cancer deaths [2].

Cutaneous metastases in breast cancer are uncommon, and scalp metastases are considered an even rarer finding [1]. Here we present a case of metastatic breast cancer in a 47-year-old female patient who, after a modified radical mastectomy, multiple lines of chemotherapy and radiotherapy, ended up developing cutaneous metastases, including metastases on the scalp, with no signs of organ failure.

## Case history

A 47-year-old female Middle-Eastern patient presented to the King Hussein Cancer Center (KHCC) in September of 2022 for further treatment of her metastatic breast cancer, which was diagnosed and initially treated in multiple facilities outside Jordan.

The patient did not smoke or drink alcohol, and suffered from supraventricular tachycardia since 2021, which was controlled using bisoprolol. She was married and had two sons and one daughter at the time of presentation. The patient did not have any psychiatric issues before her presentation at KHCC. During hospitalization, however, she suffered from major depressive disorder, which manifested in low mood, decreased appetite, fatigue, generalized weakness, and anhedonia without suicidal ideation. Additionally, she had never used any anti-depressant or anti-psychotic medications.

She was diagnosed with stage III left invasive ductal carcinoma in 2017, with no distant metastases, on the basis of imaging. Immunohistochemistry (IHC) of the breast biopsy was positive for estrogen receptor (ER) (95%), progesterone receptor (PR) (5%), and demonstrated zero score for human epidermal growth factor receptor 2 (HER-2/neu). She was treated by modified radical mastectomy in 2017, followed by 25 sessions of adjuvant radiotherapy to the left breast and tamoxifen as a hormonal therapy until 2020. Following that, the images showed left axillary lymph node enlargement and metastases to the spine, ribs, liver, and both lungs. At that time, she was started on paclitaxel-fulvestrant-letrozole, but follow-up images showed further disease progression in the known metastases, so her regimen was switched to gemcitabine-carboplatin and alpelisib in February 2021. However, in 2021, the patient suffered from progressive refractory back pain in response to analgesia, and it radiated to the left thigh. Spine magnetic resonance imaging (MRI) showed multiple progressive metastatic lesions in the spine, for which she received a number of palliative external beam radiotherapy (EBRT) sessions, which significantly contributed to relieving the pain. Afterwards, she received vinorelbine-capecitabine as palliative chemotherapy, but disease progression continued nonetheless. The patient did not undergo scalp cooling while receiving chemotherapy. Her last chemotherapy session was on 5 June 2022.

**Fig. 1:**
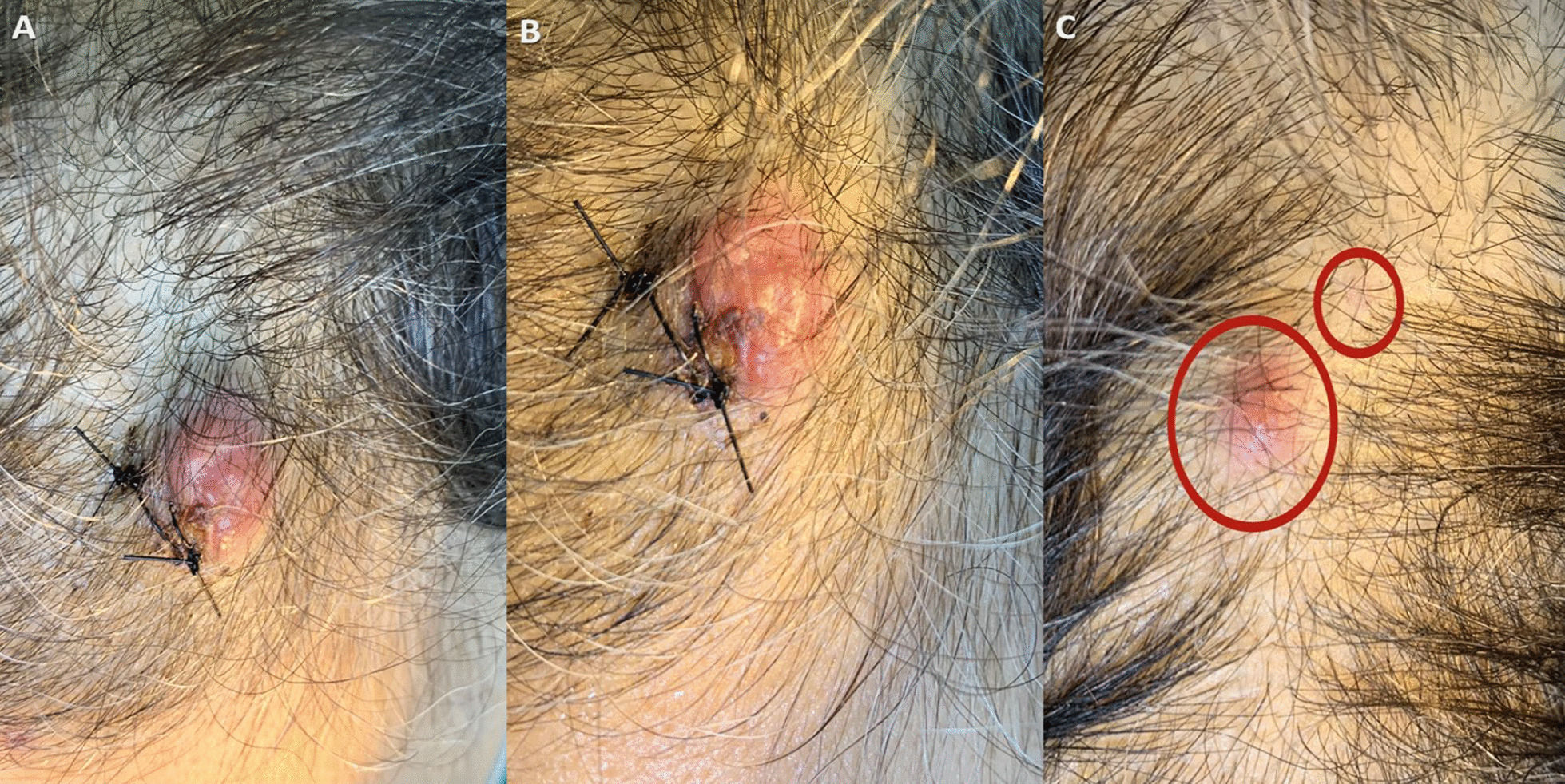
**A**, **B** The top left sided scalp mass of a 47-year-old female patient. **C** The two left parietal masses of a 47-year-old female patient. Red circles showing the two other lesions in the left parietal region; measuring approximately 2cm x 1cm x 5mm and a few millimeters, respectively

The patient presented to KHCC on 15 September 2022 seeking treatment with new lines of chemotherapy, for which she was not eligible. In addition, she only complained about enlarging scalp nodules that she had noticed 2 months prior to presentation, with no signs of multiple organ failure. They were itchy, slightly painful, and rapidly growing, especially over the 3 weeks immediately preceding her visit. Physical examination revealed three scalp nodules, the largest of which was in the left frontal area measuring approximately 4 cm × 3 cm × 0.5 cm (Fig. 1A, B), with the two other lesions in the left parietal region measuring approximately 20 mm × 10 mm × 5 mm, and a few millimeters, respectively (Fig. 1C). They were erythematous, firm, non-mobile, and non-tender. The surface was irregular, and there was no visible discharge upon applying pressure.

She also noticed a palpable mass on the central chest. It was painless, non-pruritic, and growing gradually over the last month. Physical examination revealed a mass on the mid-chest over the manubrium measuring approximately 7 cm × 5 cm × 3 cm. It was irregular, immobile, firm, and non-tender. The overlying skin was intact, with no visible redness or discharge. The impression was a metastatic lesion; however, a biopsy was not taken at the time of examination.

**Fig. 2:**
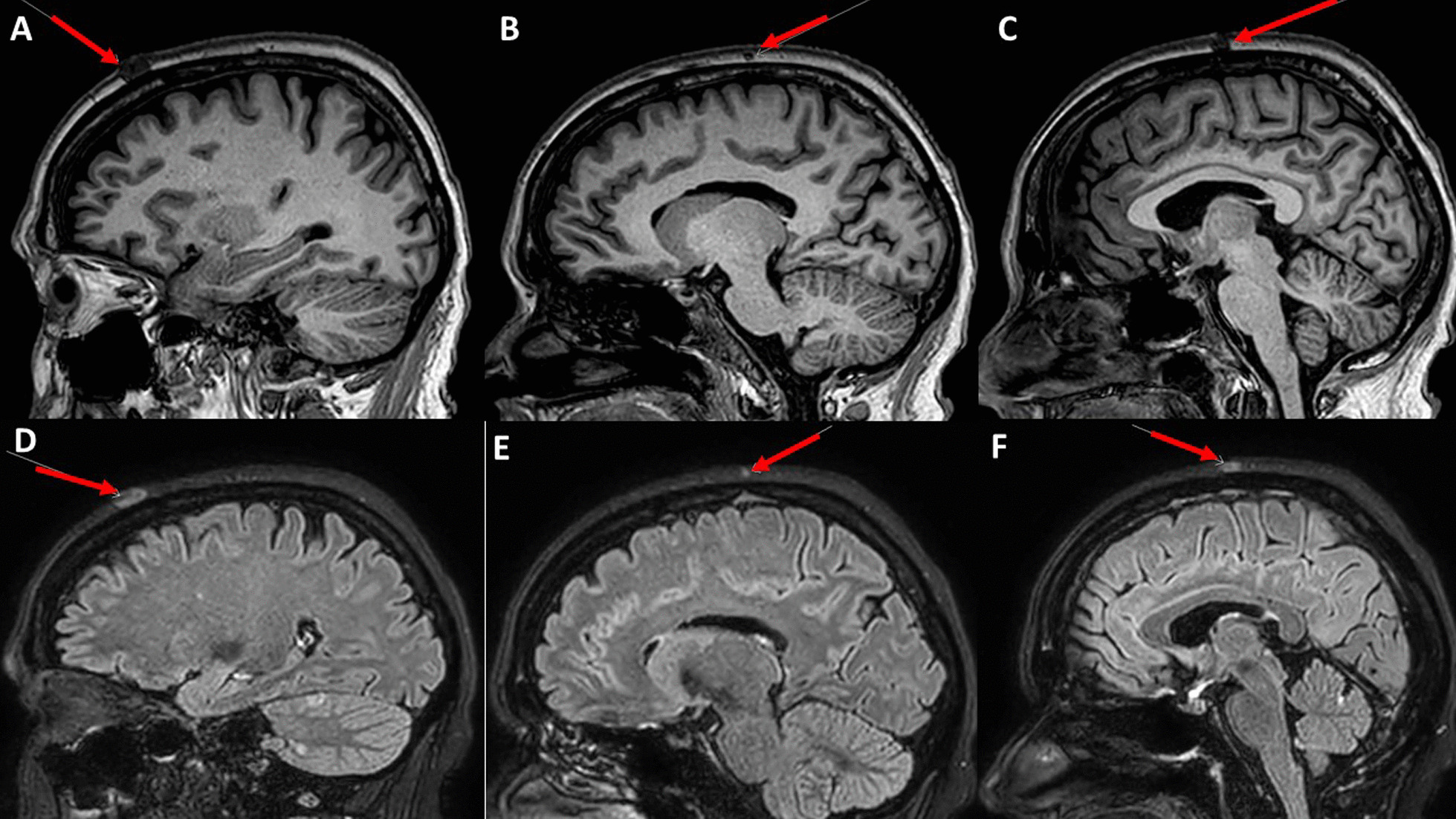
Selected sagittal images from brain MRI of T1 non-contrast and fluid-attenuated inversion recovery (FLAIR) post-contrast sequences showing three scalp lesions. **A**–**C** showed hypointense lesions on T1 indicated by red arrows. **D**–**F** showed hyperintense lesions on FLAIR. Lesions are indicated by red arrows. **A** & **D** showing the largest one on the left frontal region measures about 1.4 cm, 1cm, 0.3 cm at the time of examination.

**Fig. 3:**
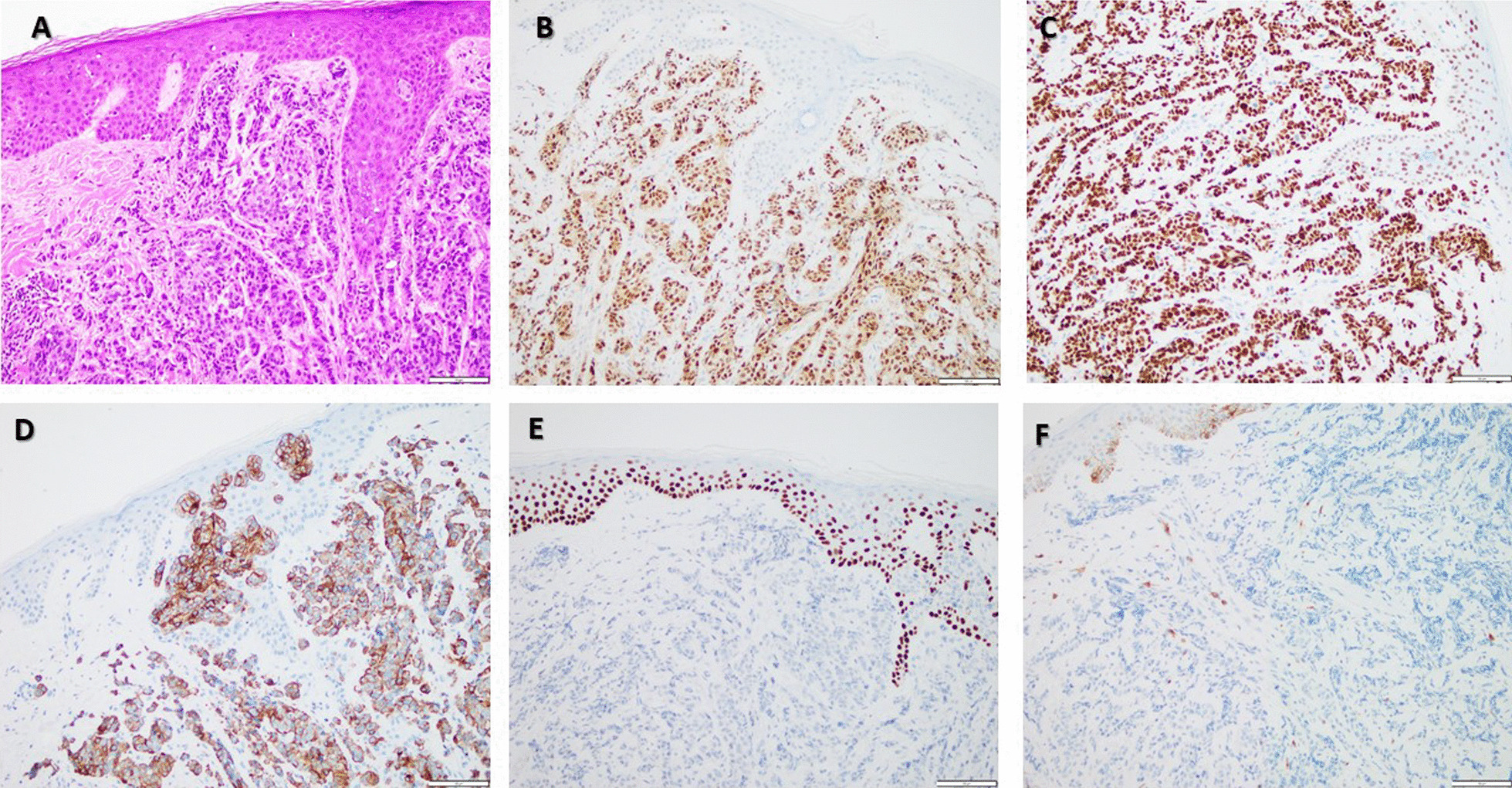
Metastatic invasive ductal carcinoma to the scalp. Hematoxylin and eosin stain of the tumor showing poorly formed glands filling the dermis with normal overlying epidermis (**A**), immunohistochemistry (IHC) for estrogen receptor (ER) showed that almost all tumor cell nuclei are estrogen receptor positive, highlighted by brown nuclear staining (**B**), IHC for GATA-3 illustrates the strong positive nuclear staining (brown) in all tumor cells (**C**), cytokeratin-7 IHC demonstrates positive membrane staining of the tumor cells (**D**), p63 IHC is negative in the tumor cell nuclei (lack of brown staining), whereas the nuclei of the overlying epidermis are positive (**E**), and IHC for CD117 is negative in the tumor cells, but shows reactivity in the melanocytes in the overlying epidermis (**F**). Original magnification of all pictures was × 200

**Fig. 4:**
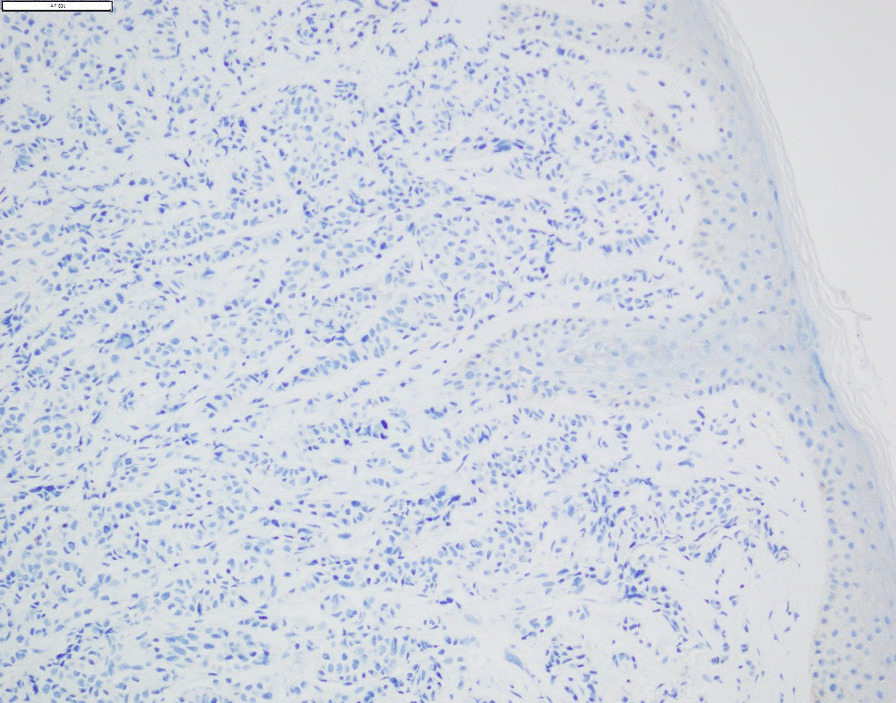
IHC for HER-2 score was negative in the tumor cells. Original magnification of all pictures was × 200

Selected sagittal images from brain MRI showed three scalp lesions (Fig. 2). A punch biopsy was acquired from the largest scalp lesion on 15 September 2022, and showed metastatic invasive ductal carcinoma with positive ER (95%), PR (5%), cytokeratin-7 (CK-7), and GATA binding protein 3 (GATA-3). P63 and KIT (CD117) were negative in the tumor cells (Fig. 3) and HER-2/neu score was zero (Fig. 4).

The patient did not receive any treatment for scalp metastases as she suffered from a chest infection and bilateral malignant pleural effusion that was proven by cytology, which progressed to septic shock before she passed away.

## Discussion

Around 6% of female breast cancer cases will present with *de novo* metastatic lesions [3]. Internal malignancies can metastasize through different routes, including the hematogenous route, lymphatics, or via direct extension from the primary tumor [4, 5].

The most common sites of metastasis in breast cancer are the lungs, bone, liver, and brain [6]. Although skin metastases from internal malignancies are rare, breast cancer in women is known to cause skin metastasis in the overlying skin or the skin proximal to the primary tumor in about 25% of breast cancer cases. These metastases are commonly found after diagnosing the patient with cancer. In some cases, however, they might be the first sign of occult visceral malignancies [7, 8].

Cutaneous metastases are commonly found in the late stages of lung, gastric, and breast cancer [9]. Scalp tumors are rare and account for only 2% of all skin tumors. However, the scalp region accounts for 4–6.9% of all cutaneous metastases, as this region has an abundant blood supply [10]. Scalp metastases indicate a sign of progression and unfavorable prognosis [3]. Chuang et al. studied patients with scalp lesions and found that 12.8% of these lesions were metastatic. He found that lung cancer was the most common primary tumor, followed by colon liver, and breast cancer, respectively, making breast cancer the least common accounting for around 7.84% [6, 11].

Cutaneous metastatic breast cancer must be distinguished from a wide variety of other neoplasms using histology. From a histopathologic point of view, a primary skin adnexal (sweat gland) adenocarcinoma is the main differential diagnosis for a subcutaneous adenocarcinoma of unknown etiology. Because the treatment and prognosis in these two entities are vastly different, a definitive diagnosis is essential [12].

Sebaceous differentiation can occur in a variety of morphologic types of breast carcinomas, including infiltrating or invasive ductal carcinomas, adenoid cystic carcinomas, and others [13]. Because lesions cannot be examined as entire three-dimensional entities in small biopsy specimens, suspected lesions should be completely excised [7].

Because primary sweat gland adenocarcinomas can express estrogen receptors, its utility in such scenarios is limited [14]. Most breast carcinomas are positive for CK-7 and negative for CK20 [15], although cases of CK-7-negative breast carcinomas can be seen in common practice. GATA-3 has gained popularity as a sensitive marker for breast carcinoma in the breast cancer literature; nevertheless, it lacks specificity and should be used in concert with other markers [16]. Since breast carcinomas are frequently negative or only focally positive for p63, a homolog of p53, whereas adnexal carcinomas are usually strongly and diffusely positive for this marker, it can be beneficial in such scenarios [12].

Some data have shown that elevated KIT (CD117) expression rarely occurs in breast cancer. KIT protein is expressed normally in breast epithelium, and hence, KIT positive breast cancers might not reflect "KIT upregulation.” Combined overexpression of c-kit and p63 indicates benign breast lesions. In contrast, there is reduced expression of c-kit in *in situ* and invasive breast carcinomas, with simultaneous overexpression in stromal cells [17, 18]. On the other hand, the expression of CD117, evaluated by IHC, was found to be specific for adnexal skin tumors with apocrine/eccrine and sebaceous differentiation [19]. As no single unique marker has been established in the differentiation of breast carcinoma and cutaneous adnexal tumors, a panel of IHC stains should always be used [3]. Table 1 summarizes the IHC stains used in our case.

**Table 1:** Summary of IHC in tumor cells of scalp lesion

ER	95%
PR	5%
HER-2/neu	Negative
GATA-3	Positive in tumor cells
CK-7	Positive
P63	Negative
KIT (CD117)	Negative in the tumor cells, but shows reactivity in the melanocytes

*ER* Estrogen receptor, *PR* Progesterone receptor, *HER2/neu* human epidermal growth factor receptor 2, *GATA3* transcription factor of the GATA family, *CK-7* cytokeratin-7, *P63* Tumor protein 63, *KIT (DC117)* receptor tyrosine kinase protein/ cluster of differentiation-117

After reviewing the literature, we found that some cases of scalp metastases were discovered years after treatment of the primary breast cancer, while others were the earliest manifestation of a metastatic occult breast cancer. Abdelhafeez AAM brought up a case of a 59-year-old woman, who had been clinically diagnosed with stage IIIA invasive ductal breast cancer in August 2015. She was treated with neoadjuvant chemotherapy and breast conservative surgery, followed by adjuvant chemotherapy and hormonal therapy. Two years later, she developed a slowly growing painless scalp nodule with histopathology suggestive of poorly differentiated adenocarcinoma [5]. Müller et al. reported a 61-year-old Caucasian woman who presented with a scalp lesion that was initially misdiagnosed as a primary cutaneous cancer with sebaceous differentiation. Only after reviewing her personal medical history, she mentioned a history of poorly differentiated invasive solid ductal breast carcinoma. Histopathologic examination of the scalp tumor revealed cutaneous metastases of a bilateral ductal breast carcinoma [7].

Alizadeh et al. reported a case of a 44-year-old female with a previous history of benign breast cyst who presented with a painless scalp mass. Pathologic examination confirmed metastatic adenocarcinoma. The IHC results revealed a pattern that favored metastatic carcinoma of a primary breast tumor [8]. Kayak et al. mentioned a 58-year-old female patient presenting with hemorrhagic lesions in the scalp, found in conjunction with a palpable left breast mass. Further work up and staging showed diffuse visceral metastases. Histopathology findings were consistent with infiltrative ductal carcinoma [20]. In Abrams’ report for breast carcinoma, the skin was only the eighteenth most common metastatic site. Inflammatory carcinoma was the most common type of metastatic carcinoma from the breast to the skin, and only rarely was it an adenocarcinomatous pattern. These findings were in contrast to our case of invasive ductal carcinoma [20].

Since alopecia is a well-known adverse effect of chemotherapy, it is worth mentioning that since the 1970s, scalp cooling has been used to prevent chemo-related alopecia. The concept was derived from the vasoconstriction brought on by scalp cooling, which reduced the amount of blood that entered the scalp, while also reducing the metabolic rate in hair follicles [21]. There were concerns about the risk of recurrence when using scalp cooling, as stem cells or malignant cells that could be present at the time of treatment would not be treated sufficiently [21]. According to the systemic review and metaanalysis of 24 full-text articles on patients with early-stage breast cancer who were receiving chemotherapy, there was no evidence that scalp cooling had increased the risk of scalp metastasis [21]. Van den Hurl came to the same conclusion that a small percentage of chemotherapy patients, whether they underwent scalp cooling or not, were at risk for developing metastases [22].

## Conclusion

Breast cancer metastasis to the scalp is an extremely rare phenomenon, which if present might be the only symptomatic sign of disease progression or widespread metastatic disease. Knowing that the patient had widespread metastases to other organs, the first impression for any patient presenting with scalp nodules would be metastases; however, a biopsy with a panel of IHC would be the only approach to distinguish them from skin tumor. In some cases, if the biopsy shows skin adnexal tumor, the management could be changed, such as considering topical chemotherapy or local resection, depending on the patient’s status.

## Data Availability

Not applicable.

## Abbreviations

CK: Cytokeratin
ER: Estrogen receptor
GATA-3: GATA binding protein 3
HER-2: Human epidermal growth factor receptor 2
IHC: Immunohistochemistry
MRI: Magnetic resonance imaging
PR: Progesterone receptor
